# Interaction of Perceptual Grouping and Crossmodal Temporal Capture in Tactile Apparent-Motion

**DOI:** 10.1371/journal.pone.0017130

**Published:** 2011-02-23

**Authors:** Lihan Chen, Zhuanghua Shi, Hermann J. Müller

**Affiliations:** 1 Department of Psychology and Key Laboratory of Machine Perception (Ministry of Education), Peking University, Beijing, P. R. China; 2 Department Psychologie, Ludwig-Maximilians-Universität München, München, Germany; 3 School of Psychological Science, Birkbeck College, University of London, London, United Kingdom; University of Sydney, Australia

## Abstract

Previous studies have shown that in tasks requiring participants to report the direction of apparent motion, task-irrelevant mono-beeps can “capture” visual motion perception when the beeps occur temporally close to the visual stimuli. However, the contributions of the relative timing of multimodal events and the event structure, modulating *uni*- and/or *cross*modal perceptual grouping, remain unclear. To examine this question and extend the investigation to the tactile modality, the current experiments presented tactile two-tap apparent-motion streams, with an SOA of 400 ms between successive, left-/right-hand middle-finger taps, accompanied by task-irrelevant, non-spatial auditory stimuli. The streams were shown for 90 seconds, and participants' task was to continuously report the perceived (left- or rightward) direction of tactile motion. In Experiment 1, each tactile stimulus was paired with an auditory beep, though odd-numbered taps were paired with an asynchronous beep, with audiotactile SOAs ranging from −75 ms to 75 ms. Perceived direction of tactile motion varied systematically with audiotactile SOA, indicative of a temporal-capture effect. In Experiment 2, two audiotactile SOAs—one short (75 ms), one long (325 ms)—were compared. The long-SOA condition preserved the crossmodal event structure (so the temporal-capture dynamics should have been similar to that in Experiment 1), but both beeps now occurred temporally close to the taps on one side (even-numbered taps). The two SOAs were found to produce opposite modulations of apparent motion, indicative of an influence of crossmodal grouping. In Experiment 3, only odd-numbered, but not even-numbered, taps were paired with auditory beeps. This abolished the temporal-capture effect and, instead, a dominant percept of apparent motion from the audiotactile side to the tactile-only side was observed independently of the SOA variation. These findings suggest that asymmetric crossmodal grouping leads to an attentional modulation of apparent motion, which inhibits crossmodal temporal-capture effects.

## Introduction

Apparent motion is a common perceptual phenomenon in our daily life. For example, two brief flashes of light separated in both time and space create an illusion of movement from the location of the first flash to that of the second flash when the spatiotemporal display parameters are within appropriate ranges [1]. Apparent motion has been observed in the visual, auditory, and tactile modalities, given the respective physical stimuli. A number of studies have shown that apparent motion in a particular modality may be influenced by static or dynamic events in another modality [2]–[4]. For example, the direction of auditory motion in one direction can be *captured* by concurrent visual motion in a conflicting direction; by contrast, the perceived direction of visual motion is not affected by incongruent auditory motion [4]. Recent work on crossmodal temporal integration has also shown that apparent motion in one modality can be modulated solely by the timing of events in another modality [5], [6]. For example, using a visual apparent-motion paradigm, Freeman and Driver [5] found that, in a repeated two-flash visual apparent-motion stream with equal inter-flash intervals (for which, when presented alone, the perceived motion direction would be ambiguous), auditory beeps slightly lagging or leading the flashes strongly influenced the perceived direction of visual motion - even though the beeps themselves did not provide any spatial information. Following the modality precision hypothesis [7], [8], on which the sensory modality with the highest temporal acuity dominates the perception of events in other modalities, Freeman and Driver attributed their results to the timing of the beeps influencing the perceived timing of the visual stimuli. Similar audiovisual temporal interactions have also been found in temporal-order judgment tasks and replicated in a number of other studies. Such influences have been referred to as ‘temporal ventriloquism’ effect, that is: when auditory and visual stimuli occur slightly asynchronously, the visual stimulus is *pulled* (being captured) into temporal alignment with the auditory stimulus [9]–[12].

Although crossmodal temporal capture has now been demonstrated in a number of studies using the apparent-motion paradigm (as noted above), whether and how this effect is mediated by perceptual grouping – *within* and *across* modalities – remains unclear. A number of unimodal (within-modality) grouping principles, including spatial/temporal proximity, similarity, and ‘common fate’, have been revealed in classical Gestalt psychology [13], [14]. For example, stimuli that are spatially and/or temporally close to each other, or that share common features, are often perceived as forming a coherent “whole”. More recently, perceptual grouping has been shown to be an important factor in crossmodal perception [15]. For example, intramodal grouping and segregation of sound pairs can enhance the segregation and discrimination of concurrent visual events [16]–[18] and bias visual temporal-order judgments [19]. However, the role of perceptual grouping in visual apparent motion is still controversial. For instance, in a control experiment, Freeman and Driver (2008) manipulated *intra*modal auditory grouping by using evenly alternating high- (H) and low-pitch (L) beeps (i.e., HHLLHH…). They found auditory grouping based on pitch alternation to have little influence on visual apparent motion, from which they concluded that audiovisual temporal integration (the temporal-ventriloquism effect) was not due to unimodal (auditory) perceptual grouping. However, evidence from other studies shows that perceptual grouping can influence crossmodal temporal interactions in perceived motion [6], [19], [20]. For example, Bruns and Getzmann found that either a continuous sound filling in the gap between two light flashes or a short sound intervening between two flashes enhanced reports of continuous visual motion, while there was no such enhancement when the sound was part of a tone sequence that allowed for intramodal (auditory) grouping *prior to* the multisensory integration of the audiovisual stimuli. Bruns and Getzmann argued that auditory events that intervene between two flashes induce the impression of a single, multimodal moving object. In a more recent study, Shi et al. [6] used visual Ternus apparent motion coupled with auditory events. In Ternus apparent motion, participants are presented with a sequence of visual frames each consisting of two horizontally arranged dots that are shifted forth and back by the inter-dot distance in successive frames. Depending on the inter-frame interval, this stimulus gives rise two alternative motion percepts: either ‘group motion’, where both dots are seen to be moving (long intervals), or ‘element motion’, where only the ‘outer’ dot is seen to be moving while the ‘inner’ dot appears stationary (short intervals). Using this paradigm, Shi et al. demonstrated that merely presenting a single sound near the first or the second visual frame did not give rise to a crossmodal temporal-ventriloquism effect; more technically, single sounds had little effect on the transition threshold between element and group motion percepts. By contrast, crossmodal temporal integration was evident with fully paired audiovisual stimuli, that is, when a sound event occurred closely in time with each visual frame.

It is important to note that the perceptual groupings implicated in the above studies fall in the categories of either *uni*modal grouping (e.g., auditory grouping based on common pitch or temporal proximity) or *cross*modal (audiovisual) grouping. Both types of perceptual grouping may influence the effects examined in the above studies. Moreover, to date, the modulatory influence of perceptual uni- and, respectively, crossmodal grouping on crossmodal temporal integration has never been systematically compared within one study. On this background, the present study, employing a directionally ambiguous tactile apparent-motion stream with different embedded auditory events, was designed to explore how perceptual grouping influences crossmodal temporal capture (temporal-ventriloquism effect).

Our motive for using the audiotactile modalities is twofold. First, we aimed to examine the crossmodal temporal interaction between two modalities with similarly high temporal acuity (i.e., the auditory and tactile modalities) [21], [22]; thus, the present study was expected to extend upon previous conclusions largely based on the use of paradigms with asymmetric temporal sensitivities, and to augment reliability-based theories of multisensory integration [23], [24]. Second, crossmodal temporal integration has, as yet, not been examined systematically with tactile apparent motion (especially movement over an extended, 90-second period of time); thus, the present study was meant to enhance our understanding of crossmodal temporal integration related to the tactile modality.

**Figure 1:**
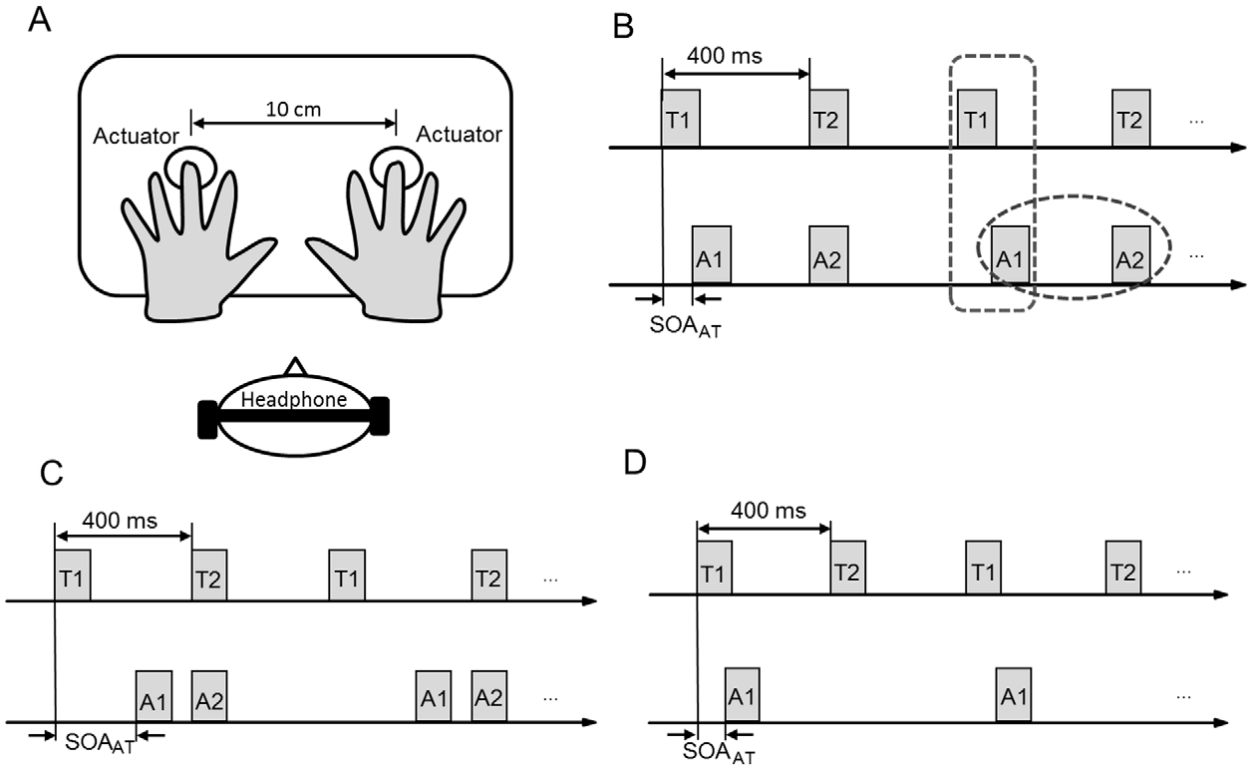
Experimetal set-up and temporal configurations of audiotactile events. (A) Illustration of the experimental setup. (B) Asynchronous and synchronous audiotactile stimulus pairs were alternated in a 90-second audiotactile stream. The SOA between tactile stimuli was consistently 400 ms. The SOA between asynchronous audiotactile stimulus pairs (SOA_AT_) was varied from −75 ms to 75 ms across trials; positive values mean the auditory beep is lagging the corresponding tactile tap. The dashed ellipse signifies unimodal auditory grouping, and the dashed rectangle crossmodal audiotactile grouping. (C) Relative to condition (b), odd-numbered beeps were temporally shifted towards even-numbered tactile taps. The odd-numbered audiotactile SOA_AT_ was set to 325 ms. (D) Auditory beeps were paired only with the taps from the initial side (either the left or the right). The audiotactile SOA_AT_ varied from −75 ms to 75 ms across trials.

In our paradigm, participants placed the tips of their left and right middle fingers on the surface of two tactile actuators (one on the left and one on the right side), while wearing headphones. The two tactile actuators produced alternating taps at a rate of 2.5 Hz for 90 seconds; concurrently, a train of mono-beeps was paired with the stream of tactile taps (for details, see Methods and Figure 1). After an initial presentation for 4 seconds, participants started to hold one foot pedal (the left or the right one) pressed to indicate their perceived direction of tactile motion; they were instructed to switch to the other foot pedal as soon as they perceived the motion direction to be reversed. In this way, it was possible to measure the (phase) durations of apparent motion in one or the other direction.

In order to examine the influence of uni- and crossmodal grouping on crossmodal temporal integration, we varied the auditory-auditory interval and the audiotactile interval separately. In more detail, to modulate unimodal (intra-auditory) grouping (see dashed ellipse in Figure 1B), we presented either interleaved short and long auditory intervals or equal auditory intervals within the stream of audiotactile stimuli. And to modulate crossmodal grouping (see dashed rectangle in Figure 1B), we varied the audiotactile pairing, along with the audiotactile stimulus onset asynchronies (SOAs).

Experiment 1 was designed to establish crossmodal (audiotactile) temporal integration in tactile apparent motion. Analogously to the paradigm of Freeman and Driver [5], we introduced configurations of full (i.e., one-to-one) pairing audio-tactile stimuli: each tactile tap paired with one beep, where even-numbered beeps were always synchronous with the onsets of the tactile taps on one side and odd-numbered beeps were asynchronous, by a given SOA (−75, −50, −25, 0, 25, 50, 75 ms), with the onsets of the tactile taps on the other side (see Figure 1B). The results revealed a crossmodal (auditory-on-tactile) temporal-capture effect similar to the auditory-on-visual effect reported by Freeman and Driver.

In Experiment 2, we went on to examine the influence of crossmodal grouping on the crossmodal temporal interaction established in Experiment 1, by comparing the influence of an audiotactile SOA of 75 ms (Figure 1B; full-pairing event configuration) with that of 325 ms (Figure 1C; shifted full-pairing configuration). In both conditions, the shorter of the two auditory intervals (between A1 and A2) is pairing the odd-numbered interval between tactile taps (T1-T2, see Figure 1B and 1C). Given this, one would expect the influence of *uni*modal auditory grouping (between A1 and A2) on tactile apparent motion to work in the same direction in both audiotactile SOA conditions (depicted in Figures 1B and 1C, respectively). However, with the audiotactile SOA of 325 ms, *cross*modal grouping between auditory and tactile events would take place asymmetrically around even-numbered (T2) taps, compared to the more balanced grouping around odd-numbered and even-numbered taps in the 75-ms SOA condition. Thus, if *cross*modal grouping influenced the temporal capture effect, one would expect differential modulations of tactile apparent motion between the two conditions (as a baseline, a synchronous audiotactile condition, with an SOA of 0 ms, was also included in Experiment 2). The results revealed the direction of the temporal-capture effect to be reversed with the extended audiotactile SOA of 325 ms, compared to the 75-ms SOA, suggestive of an influence of *cross*modal grouping.

Finally, in Experiment 3, we omitted the synchronous beeps, while varying the SOA of the asynchronous audiotactile pairs, in order to further examine the interaction between crossmodal grouping and crossmodal temporal integration (see Figure 1D). With this manipulation, auditory beeps were paired only with one side (either the left or the right) of tactile taps (which is why we refer to this condition as ‘half-paring’). If balanced crossmodal grouping is *not* a precondition for the crossmodal temporal interaction, one would expect the results of Experiment 3 (half-pairing condition) to be similar to those of Experiment 1 (full-pairing condition), since the audiotactile SOAs were the same. Alternatively, if asymmetric crossmodal grouping competes with crossmodal temporal capture, one would envisage differential outcomes between the full and the half-paring conditions (realized in Experiments 1 and 3, respectively): the full-pairing audiotactile stream would be subject to a crossmodal temporal-capture effect (as actually observed in Experiment 1); by contrast, the half-pairing condition (realized in Experiment 3) would show little influence of the auditory timing due to the incomplete grouping of the auditory with the tactile events, analogously to the results of audiovisual temporal-ventriloquism study [6], [11]. Experiment 3 failed to reveal a significant influence of the audiotactile SOA, consistent with crossmodal temporal capture being prevented under the half-pairing condition; however, apparent motion was subject to a ‘global’ (i.e., SOA-independent) biasing effect: there was a strong tendency for perceiving motion from the audiotactile side to the tactile-only side. After detailing the results (see Results section below), the implications of this set of findings finding will be developed in the Discussion.

## Results

In bistable perception, participants often show a strong (but transient) bias initially for reporting the percept of the first presentation (i.e., in the present experiments, the direction indicated by the sides of the first two taps) [25]. To reduce such initial biases in the present experiment, response recording commenced only four seconds after the start of the audiotactile stimulus stream. To further disassociate any initial preference from an influence of auditory timing, the responses of left- and rightward tactile apparent-motion directions were recoded in terms of “initial direction” (i.e., perceived direction congruent with the direction indicated by the first two taps) and “reverse direction” (opposite to the “initial direction”) and, accordingly, the pedal press times (i.e., *phase durations*) were collected and calculated separately for the “initial” and the “reverse” directions in each audiotactile condition. Since the phase durations often have the same intra-participant distribution, but vary substantially among participants [25], [26], the phase durations were normalized for each of the participants relative to their respective means.

### Experiment 1. Tactile apparent motion with a full pairing audiotactile stream

**Figure 2:**
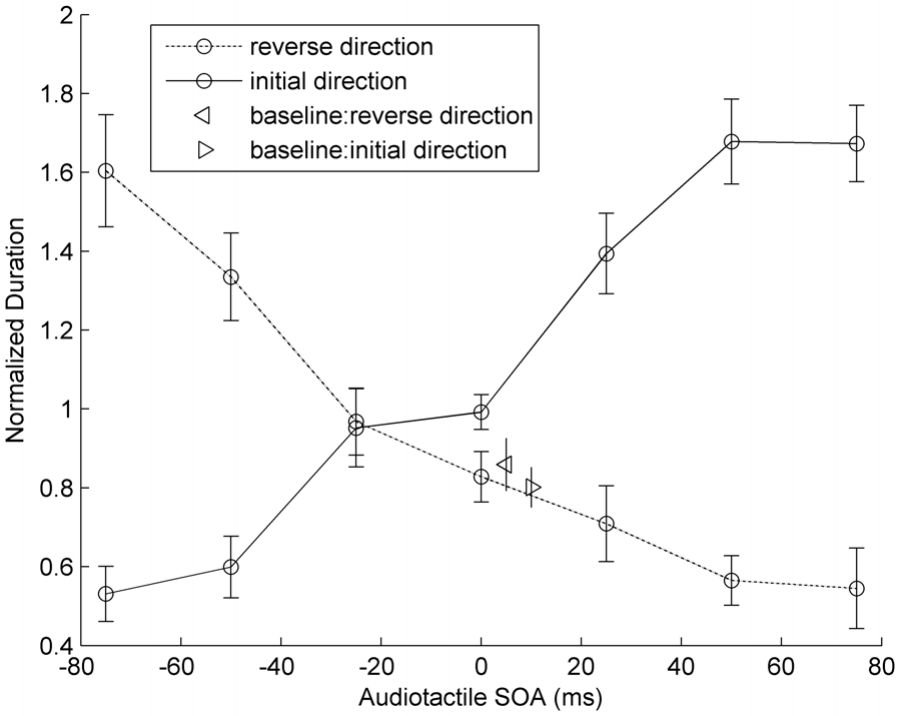
Normalized phase durations of tactile apparent motion in Experiment 1. Normalized phase durations (and associated standard errors) of tactile apparent motion as a function of audiotactile SOA with a full-pairing audiotactile stream. The solid line represents mean phase durations for the “initial direction”, the dotted line those for the “reverse direction”. The audiotactile asynchronies systematically influenced the direction of the tactile apparent motion. For the “without-sound” baseline conditions, the rightward-pointing triangle denotes responses of “initial direction”, and the leftward-pointing triangle responses of “reverse direction”.

Figure 2 shows the mean normalized phase durations for the two types of responses as a function of the audiotactile SOA. A pairwise *t*-test showed that in the baseline condition (without sounds), the phase durations for the two types of responses (i.e., “initial direction” and “reverse direction”) did not differ significantly from each other, *t*(10) =  −1.322, *p* = 0.216, indicating that the initial bias had dissipated after four seconds of stimulus presentation However, there remained a marginal initial bias after four seconds for tactile apparent motion in the synchronous audiotactile stream (SOA  = 0 ms), *t*(10) = 2.179, *p* = 0.054. For the conditions with sounds present, Figure 2 shows a clear audiotactile interaction in the perceived tactile motion across the different audiotactile SOAs. We selected the phase durations of “initial-direction” responses for further analysis of the auditory capture effect (the results would be analogous for the “reverse direction”). A repeated-measures ANOVA revealed a significant main effect of auditory timing, *F*(6,60) = 28.534, *p*<0.001, and a linear contrast test showed that the phase duration increased linearly with increasing audiotactile SOA, *F*(1,6) = 167.289, *p*<0.001. This indicates that asynchronous auditory-tactile timing did indeed influence tactile apparent motion, with the influence being systematic and bidirectional. For example, an audiotactile SOA of 50 ms (when the odd numbered beeps lagged the corresponding taps by 50 ms) produced a dominant percept of “initial direction”, while an SOA of −50 ms gave rise to a dominant percept of “reverse direction”. Note that the opposite trends with respect to “initial direction” and “reverse direction” crossed at the audiotactile SOA of −25 ms (rather than the SOA of 0 ms). This slight asymmetry may be attributable to a shift in audiotactile simultaneity resulting from temporal recalibration and adaptation in the extended (and repeated) audiotactile stream [27], [28], or the small difference between the auditory and tactile stimulus durations used in the experiment. However, the general trends are consistent with Freeman and Driver's [5] ‘audiovisual’ study, where auditory timing was found to influence visual apparent motion in a similar way.

### Experiment 2. Tactile apparent motion with a shifted full pairing audiotactile stream

**Figure 3:**
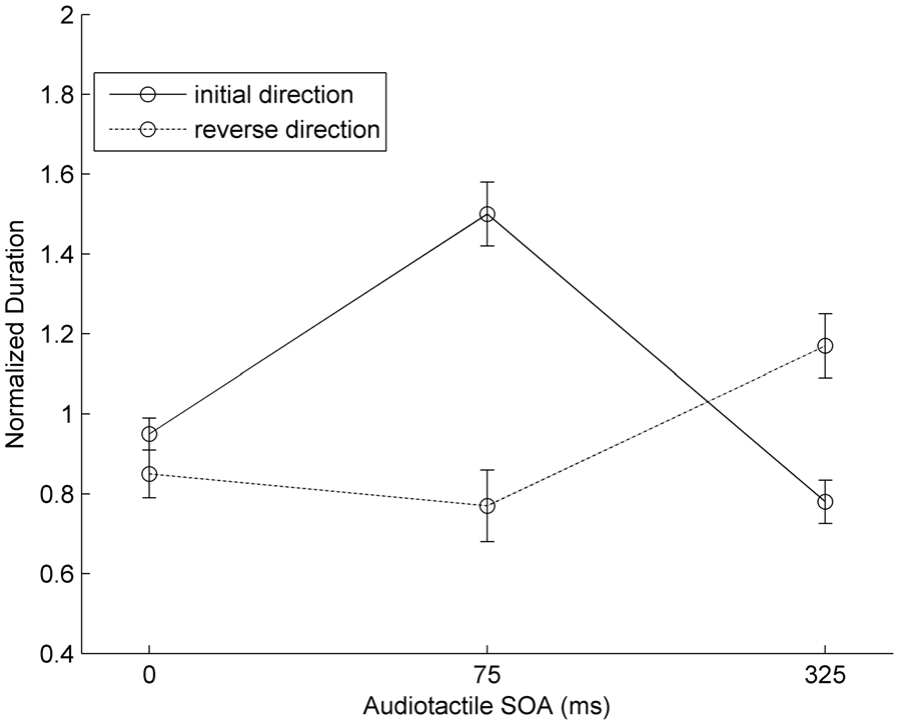
Normalized phase durations of tactile apparent motion in Experiment 2. Normalized phase durations (and associated standard errors) of tactile apparent motion as a function of audiotactile SOA with a shifted full-pairing audiotactile stream.

Figure 3 presents the mean phase durations for “initial-direction” and “reverse-direction” responses as a function of the (variable) audiotactile SOA. A repeated-measures ANOVA for the “initial-direction” responses revealed the main effect of audiotactile SOA to be significant, *F*(2,20) = 11.66, *p*<0.01 (*F*(2,20) = 7.215, *p*<0.01, for the “reverse direction”). Bonferroni-corrected pairwise comparisons showed that for both “initial-direction” and “reverse-direction” responses, the mean phase durations differed significantly between the 75-ms and the 325-ms SOA, *p*s<0.05. With an audiotactile SOA of 75 ms, the response pattern was similar to that in Experiment 1, that is, characterized by dominance of “initial direction”. However, the dominant motion direction was changed to “reverse direction” when the audiotactile SOA was increased to 325 ms. The differential dominance patterns of tactile apparent motion between these two conditions is the most interesting finding of Experiment 2, which demonstrates that crossmodal grouping can strongly influence the crossmodal temporal integration.

### Experiment 3. Tactile apparent motion with a half pairing audiotactile stream

**Figure 4:**
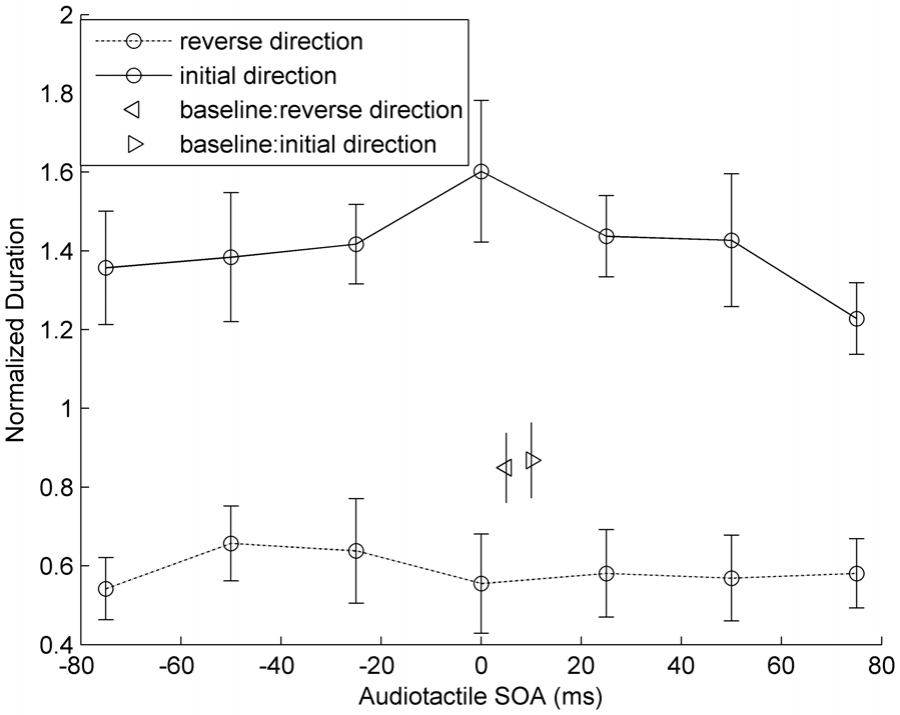
Normalized phase durations of tactile apparent motion in Experiment 3. Normalized phase durations (and associated standard errors) of tactile apparent motion as a function of audiotactile SOA with a half-pairing audiotactile stream. The solid line represents mean phase durations for the “initial direction”, the dotted line those for the “reverse direction”. Regardless of the audiotactile SOAs, a globally dominant direction of apparent motion, namely, “initial direction”, was observed. The rightward-pointing triangle denotes responses of “initial direction”, and the leftward-pointing triangle responses of “reverse direction”, for the baseline (without-sound) conditions.

Experiment 3 was similar to Experiment 1, except that the “asynchronous” beeps were omitted (they were presented in Experiment 1). The mean normalized phase durations are shown in Figure 4.

A pairwise *t*-test comparing the two perceived directions in the baseline condition (without beeps) revealed no difference, *t*(10) = 0.286, *p* = 0.781. A repeated-measures ANOVA of the phase durations for “initial-direction” responses, with the single factor audiotactile SOA, failed to reveal a significant SOA effect, *F*(6,60) = 1.069, *p* = 0.391. Likewise, there were no significant differences among audiotactile SOAs in the phase durations of “reverse-direction” responses, *F*(6,60) = 0.451, *p* = 0.841. Given this, we collapsed the phase durations across all SOAs, separately for “initial-direction” and “reverse-direction” responses, and compared the resulting values to the corresponding baseline conditions: for the “initial-direction” responses, the phase durations were significantly longer compared to the baseline, *t*(10) = 3.140, *p*<0.05; by contrast, for the “reverse-direction” responses, they were significantly shorter *t*(10) =  −3.534, *p*<0.01. Thus, in contrast to Experiment 1, “initial-direction” responses were dominant across all seven audiotactile SOAs, regardless of auditory timing (the audiotactile SOA varied from −75 ms to 75 ms). This indicates that the half-pairing auditory beeps created a “globally” dominant percept of motion direction from the side of the audiotactile stimuli to the side of the tactile-only stimuli.

## Discussion

This study examined the influences of perceptual grouping and crossmodal temporal integration of auditory with tactile events in a tactile apparent-motion stream. With a full pairing audiotactile configuration (Experiment 1), we varied the audiotactile asynchronies from −75 ms (beep leading tap) to 75 ms (beep trailing tap) in the odd numbered pairs, while keeping the even numbered pairs synchronous. We observed the (bi-stable) tactile apparent-motion rivalry (i.e., perceived motion going either left- or rightwards) to be systematically resolved by the audiotactile asynchrony. However, contrary to our original expectation, when the audiotactile asynchrony was increased (to 325 ms) such that the (asynchronous) beeps occurred temporally proximal to (i.e., “shifted” towards) the even numbered tactile stimuli, a reversed effect on the direction of apparent motion was found (Experiment 2). In Experiment 3, which used half-pairing audiotactile stimuli, a consistently dominant direction of apparent motion was observed: the dominant direction went from the location (side) with audiotactile stimulus pairings towards the location (side) with a pure tactile stimulus.

The results of Experiment 1 are consistent with Freeman and Driver's [5] finding that auditory beeps leading or lagging visual stimuli can readily bias visual apparent motion. In their study, the target modality (in which to-be-judged apparent-motion stimuli were presented) was vision, which is characterized by low temporal acuity. Our results show that apparent motion in the tactile modality, which has a high temporal resolution, can likewise be influenced by auditory timing. Both findings can be interpreted in terms of a “temporal-ventriloquism” effect [11], that is, the timing of target stimuli (in either the tactile or the visual modality) is systematically influenced by the timing of auditory beeps. In audiotactile streams, lagging odd-numbered beeps *pull* the timing of the corresponding taps closer to the subsequent, even-numbered taps, thus leading to dominant responses of “initial direction”. Similarly, leading odd-numbered beeps *push* the timing of the corresponding taps away from the subsequent taps, giving rise to the opposite dominant motion percept of “reverse direction”.

However, the temporal ventriloquism account cannot explain the results of the condition with the long audiotactile asynchrony (325-ms SOA, Experiment 2). If the timing of the asynchronous beep captured the timing of either the first or the second tactile tap, the auditory beep at the 325-ms SOA would still enhance the “initial-direction” percept, since the sound would *attract* the two taps (whether by acting on the first or the second tap) closer to each other. Similarly, based on the notion of (intramodal) auditory grouping, with both 75 and 325-ms SOAs, short intervals were paired with odd-numbered tactile intervals – so that one would also expect a dominance of “initial-direction” percepts, rather than the opposite. An alternative explanation, which assumes “bridging” two visual (i.e., by extension to the present scenario: tactile) events by an intervening auditory event [10], would predict similar results to the temporal ventriloquism or auditory-grouping accounts, namely, dominant apparent motion in the “initial direction”, for both the 75- and 325-ms SOA conditions. However, (on all these accounts) unexpectedly, the results of Experiment 2 showed exactly the opposite effect: dominant apparent motion in the “reversed direction”.

It is known that crossmodal integration takes place within a certain, limited temporal and spatial range [6], [15], [29]–[32]. On this background, in the condition with the audiotactile SOA of 325 ms, odd-numbered beeps were shifted close to the even-numbered taps, thus weakening the crossmodal grouping of the odd-numbered audiotactile stimuli (pair) and strengthening the crossmodal grouping of even-numbered stimuli (A1-T2-A2 in Figure 1C). Such asymmetric crossmodal grouping for even- and odd-numbered stimuli may cause an attention shift towards the salient taps (T2) (even though participants were told to disregard the sounds). This, in turn, would prime the following tactile events (T2-T1). This is consistent with previous studies of attentional modulations of apparent motion [33]–[35]. For example, in the study of the audiovisual or the tactile-visual line motion illusion [36], where a beep sound or an electric pulse (cue) is presented on either the left or the right side and this stimulus is accompanied or followed by a visual line presented in close proximity to the cue, the line is perceived to grow rapidly from the crossmodally stimulated side (this is referred to as the “line motion” effect). The crossmodal line motion effect has been attributed to a spatial-attentional bias induced by the auditory or tactile cue. In our case, strong crossmodal grouping on one side may similarly have served as a “cue” (even though the auditory beeps carried no spatial information), inducing one dominant motion direction.

In Experiment 3, we further examined the interaction between crossmodal grouping and crossmodal temporal interaction by removing the synchronous beeps. Although the audiotactile asynchrony was varied from −75 ms to 75 ms, as in Experiment 1, an overwhelming dominant direction of apparent motion – namely, from the audiotactile side to the tactile-only side – was found across all SOAs. That is, under these conditions, crossmodal temporal timing had no effect on tactile apparent motion. In previous studies of the temporal-ventriloquism effect using temporal-order judgments [11], [37], the sensitivity of visual temporal order judgments increased only when two visual stimuli were paired with two auditory stimuli. Analogously to the present results, a single beep failed to produce a temporal-ventriloquism effect. In a more recent study with apparent motion [6], a null effect of single sounds in audiovisual apparent motion has also been reported. Previous accounts of the absence of a temporal ventriloquism effect with single sound configurations have attributed it a violation of the “assumption of unity” [7], [8], [11]. On this assumption, crossmodal integration makes sense only when the perceptual system has evidence that the two separate multisensory events (e.g., one auditory and one visual) originate from a common source [7]. Although this assumption could explain the null effect of crossmodal temporal modulation in the half-pairing (Experiment 3) and shifted-pairing (Experiment 2) conditions, it does not predict which direction of motion prevails in these conditions. One feasible account may be derived if assuming that a ‘biased-competition’ mechanism [38], [39] is at work. The biased-competition framework assumes that when two (or more) neural assemblies compete with each other for representation, attentional biases in the system operate (over time) to make one assembly win the competition and suppress the competitor(s). Applied to the present paradigm, how an apparent-motion display is perceived depends on the relative balance of crossmodal grouping (the grouping of ‘coincident’ events in the nontarget and target modality) and crossmodal temporal capture (i.e., modulation of the timing of events in the target modality by the timing of events in the nontarget modality) – two mechanisms that may be assumed to be in competition with each other, where spatial attention may exert a biasing influence on how the competition is resolved. In the half-pairing condition realized in Experiment 3, asymmetric audio-tactile grouping on the two sides of stimulus presentation (beep plus tap on one side vs. tap only on the other side) may generate a spatial-attentional bias towards the side of the crossmodal grouping. This would make the tactile stimulus on this side more salient and afford it “prior entry”, thus giving rise to apparent tactile motion from the side of the audiotactile grouping to the other side. This is consistent with previous studies [33]–[36] that have shown attentional modulation of apparent motion to be of considerable strength, such as in the line motion illusion. By contrast, crossmodal temporal capture has been found to be a relatively weak effect [6], [19], [20]. Consequently, the latter temporal effect may be inhibited (or swamped) by the former spatial modulation.

In summary, examining tactile rivalry apparent motion dependent on different audiotactile configurations, we found a systematic influence of auditory timing on the motion percept in a full-pairing crossmodal condition. However, this temporal ventriloquism effect was abolished under conditions with half-pairing (unbalanced) and temporally shifted full-pairing configurations. Unimodal grouping based on auditory time interval or crossmodal temporal capture cannot readily explain the reversed pattern of audiotactile interaction with an audiotactile SOA of 325 ms. We propose an alternative account, namely, that unequal odd- and even-numbered audiotactile stimulus pairs leads to an attentional modulation of crossmodal grouping, which in turn prevents (or inhibits) crossmodal temporal integration. To test the hypothesis of a general attentional-saliency modulation of crossmodal temporal capture in the apparent-motion paradigm, it would be interesting to compare the present findings (tactile target modality) with conditions in which the target modality is reversed (auditory modality), that is, to examine the influence of touch modulations on auditory apparent motion rivalry.

## Materials and Methods

### Participants

Eleven paid participants participated in Experiment 1 (6 females, average age 26.6), Experiment 2 (7 females, average age 26.7), and Experiment 3 (7 females, average age 25.5). None of the participants reported any history of somatosensory disorders. They were all naïve as to the purpose of the study and were paid after the experiment. The study was approved by the Ethics Committee, Faculty of Psychology and Education, Ludwig-Maximilian University. All experiments were conducted in accordance with the guidelines of Ethical Principles of Psychologists. Written informed consent was obtained from each participant before experiments.

### Apparatus and stimuli

A customized tactile stimulus generator (Heijo Research Electronics, UK) was connected to a HP PC (AMD Athlon 64 Dual-Core processor) via the LPT port. The two solenoid actuators, which were embedded in a sponge with a fixed center-to-center distance of 10 cm, and used pulse signals to push the central pin out, producing “indentation” taps to two fingers (see Figure 1A). We conducted a pilot experiment to compare auditory capture of tactile apparent motion (as in Experiment 1) between two types of tactile stimuli produced by pulse signals of 10 ms and 30 ms, respectively. In both conditions, we found essentially the same pattern. To avoid overheating of the solenoids, the duration of a single tap was set to 10 ms and the stimulus onset asynchrony (SOA) between two successive taps was set to 400 ms. Mono-beeps (60 dB, 1000 Hz, 30 ms) were generated by an embedded high-precision M-AUDIO Delta 1010 Sound Card and delivered through a headset (RT-788V, RAPTOXX) to both ears. Participants' responses were acquired via two foot pedals. The experimental program was developed using Matlab (Mathworks Inc.) and Psychophysics Toolbox [40].

### Design and procedure

Prior to the formal experiment, participants received a practice session to become familiar with the procedure and the experimental task. They were asked to place the tips of their left and right middle fingers such as to cover the surface of the left and right tactile actuators. A trial started with a fixation cross in the center of the monitor in front of the participants, which participants were instructed to fixate throughout the trial. After a random interval of 500–1000 ms, the two tactile actuators produced alternating (finger indentation) taps with a fixed SOA of 400 ms (2.5 Hz), repeated for 90 seconds. The initial tap occurred randomly on either the left or the right middle finger (see Figure 1A). Experiment 1 comprised of seven audiotactile conditions (SOAs) and one baseline condition (without beeps), which were randomized across trials. In the audiotactile conditions, a train of beeps was paired with a train of tactile taps, where even-numbered beeps were synchronous with the onsets of the tactile taps on one side and odd-numbered beeps were asynchronous, by a given SOA (−75, −50, −25, 0, 25, 50, 75 ms), with the onsets of the tactile taps on the other side (see Figure 1B). After an initial presentation of these events for 4 seconds, a visual-cue word (“begin”) was presented in the center of the screen prompting participants to initiate their responses, that is, indicate the perceived direction of the tactile apparent motion, irrespective of the accompanying sounds. Participants were asked to hold one foot pedal pressed to indicate the perceived direction of tactile apparent motion (left foot pedal for leftward motion, right pedal for rightward motion) and to switch the foot pedal immediately when the perceived direction changed, disregarding the auditory stimuli. In the experiment, eight conditions were repeated four times, with counter-balancing of the initial motion direction. Experiment 2 was similar to Experiment 1, but only the following three audiotactile SOAs were compared: 0, 75, and 325 ms. Note that with an audiotactile SOA of 325 ms, the first beep led the second tactile tap by 75 ms, while the second beep synchronized with the second tap (thus, the auditory-auditory SOA was shorter, namely: 75 ms, in this condition; see Figure 1C). Experiment 3 was essentially the same as in Experiment 1, except that the “synchronous” beeps were removed in the audiotactile stream; that is, only odd numbered tactile stimuli were paired with sounds (Figure 1D).
